# Endoscopic and Robotic Parotidectomy for the Treatment of Parotid Tumors: A Systematic Review and Meta-Analysis

**DOI:** 10.3389/fonc.2021.748885

**Published:** 2021-11-10

**Authors:** Shanwen Chen, Mei Zhao, Dong Wang, Yi Zhao, Jianxin Qiu, Yehai Liu

**Affiliations:** ^1^ Department of Otorhinolaryngology - Head and Neck Surgery, The First Affiliated Hospital of Anhui Medical University, Hefei, China; ^2^ Oncology Department of Integrated Traditional Chinese and Western Medicine, The First Affiliated Hospital of Anhui Medical University, Hefei, China

**Keywords:** endoscopic surgery, robotic surgery, parotid gland, oncology, complication

## Abstract

**Background:**

The goal of this review was to introduce endoscopic/robotic parotidectomy (EP/RP) and compare EP/RP against conventional parotidectomy (CP) regarding the intraoperative and postoperative parameters in the treatment of parotid tumors.

**Methods:**

A systematic literature search of medical databases (PubMed, Embase, and Cochrane Central Register of Controlled Trials) was performed from inception to November 2020 to generate relevant studies.

**Results:**

A total of 13 eligible studies (572 patients) were included for systematic review, and 7 out of 13 comparable studies for the quantitative synthesis of outcomes. Patients who underwent EP were characterized by less intraoperative bleeding volume, shorter incision length, and higher satisfaction postoperatively (WMD, 95% CI, -42.80; - 58.23 to -27.37; p < 0.01; WMD, 95% CI, -5.64; -7.88 to -3.39; p < 0.01; SMD, 95% CI, 1.88; 1.46 to 2.31; p < 0.01, respectively). However, operative time and risk of facial palsy exhibited no significant differences (WMD, 95% CI, -11.17; -26.71 to 4.34; p = 0.16; OR, 95% CI,0.71; 0.39 to 1.32; p = 0.28, respectively).

**Conclusions:**

Our findings suggest that the current evidence does not adequately support EP is equally safe and effective as CP. In certain selected cases, endoscopic technology has its unique advantages. For patients with strong cosmetic needs, endoscopic or robotic techniques may be an alternative through adequate preoperative evaluations.

**Systematic Review Registration:**

International Prospective Register of Systematic Reviews, identifier CRD42020210299.

## Introduction

Benign parotid tumors account for the majority of parotid tumors (1). The preferred treatment of choice tends to be surgical resection. Currently, the majority of clinical methods are traditional “S” incisions, which can fully expose the parotid tissue while preserving important structures such as the facial nerve. Unfortunately, this conventional method will ultimately leave a large facial scar from preauricular to submandibular nodes, causing a non-negligible psychological burden and reducing the quality of life of patients (2, 3).

Additionally, modified incisions to the parotid have been used in patients with benign parotid tumors to improve the postoperative appearance. These minimally invasive approaches, however, have not been extensively used due to the high risk of structure injury (4). In the past two decades, the use of endoscopy and robotics techniques, as an emerging alternative strategy, has been demonstrated in many studies, leading to the gradual popularization and adoption of the concept of minimally invasive surgery. These procedures possess the advantages of small trauma, well exposure, and satisfying cosmetic effects. In particular, endoscopic techniques have been applied in head and neck lesions, including thyroid lesions, thyroglossal duct cysts, parapharyngeal space tumors, and even neck dissection (5–8). More importantly, it can obtain an excellent cosmetic effect on the premise of safety. Benefitting from the advantages of magnifying endoscopy, it is accessible to identify nerves and small vessels during operation. Thus, small incision approaches, including preauricular, retroauricular, hairline, or transoral have been largely developed in parotidectomy with endoscopic assistance (9, 10).

Although minimally invasive surgical techniques, particularly endoscopic-assisted parotid surgery, have been introduced more than 10 years, the progressive development of technology is less than that of thyroid. One reason is certainly the difference of incidence, the ease of use and safety of the new technology warrant consideration as well. To date, there is no systematic review summarizing the findings on this technique. In this regard, the present study aimed to perform a systematic review and meta-analysis introducing the safety and efficacy of endoscopic or robotic-assisted parotid gland surgery.

## Methods

This review was conducted following the Preferred Reporting Items for Systematic Reviews and Meta-Analyses (PRISMA) guidelines (11). Two of the authors (S.C. and M.Z.) independently searched the electronic databases including PubMed, Embase, and the Cochrane Central Register of Controlled Trials (CENTRAL) for articles of interest published before November 2020.

This study was registered in the International Prospective Register of Systematic Reviews (CRD42020210299). The search of the databases was performed by combining the Mesh terms and keywords, including “endoscopy” OR “endoscopic” OR “robotic surgical procedures” OR “robot” OR “robotic” OR” minimally invasive” AND “parotid gland” OR “parotid” OR “parotidectomy” OR “parotid surgery”. Articles that fulfilled the inclusion criteria were included in the review. The authors also reviewed the reference lists of the included studies to optimize screening and selection. All analyses were based on previous published studies; thus, no ethical approval and patient consent are required.

### Study Selection

Inclusion criteria included: (1) both randomized clinical trials (RCTs) and observational studies, (2) studies that reported the outcomes of endoscopic or robotic-assisted parotidectomy, (3) articles reported in English language, and (4) if more than one study presented data from the same study participants, either the study of the higher quality or the most comprehensive was included. Exclusion criteria included: (1) any publication that did not meet the above inclusion criteria, (2) sialendoscopy, (3) salivary calculus, and (4) conference abstracts, editorials, and case report ≤ 5.

### Data Extraction and Quality Assessment

The following data variables were extracted: first author, year of publication, country, study design, surgery approach, number of patients, gender and age, operative details, and outcomes. The main surgical outcomes included operative time, bleeding volume, incision length, cosmetic satisfaction, and facial never palsy. The secondary outcomes included drainage volume, length of hospital stay, and other complications. Finally, surgical completeness and tumor recurrence were also documented.

Further, the same authors independently assessed the quality of the included studies. The Methodological Index for Non-Randomized Studies (MINORS) scale was applied to evaluate the non-RCTs and Cochrane Collaboration tools for RCTs (12, 13).

### Data Synthesis and Analysis

When the included studies were comparable, a meta-analysis was performed, otherwise, only a systematic review would have been conducted. Cosmetic satisfaction was assessed with a visual analog scale (VAS). When necessary, we subtracted the mean from the possible extremum while keeping the standard deviation unchanged to ensure that the directionality of the variables was consistent with higher values indicating high satisfaction. Medians were converted to means using a previously described methodology (14). Review Manager program version 5.4 was applied to perform statistical data analysis. For summarized continuous data, weighted mean difference (WMD) and/or standardized mean difference (SMD) were expressed, while dichotomous variables were examined using odds ratio (OR), reported with 95% CIs. Overall results were pooled using a random-effects model based on the variation between studies. The homogeneity test among studies was analyzed using I^2^ tests, which was interpreted on the following scale: I^2^ value of 25% indicates low heterogeneity, > 50% moderate heterogeneity, and > 75% high heterogeneity. A value of p <.05 was considered statistically significant. Sensitivity analyses were carried out when appropriate.

## Results

**Figure 1:**
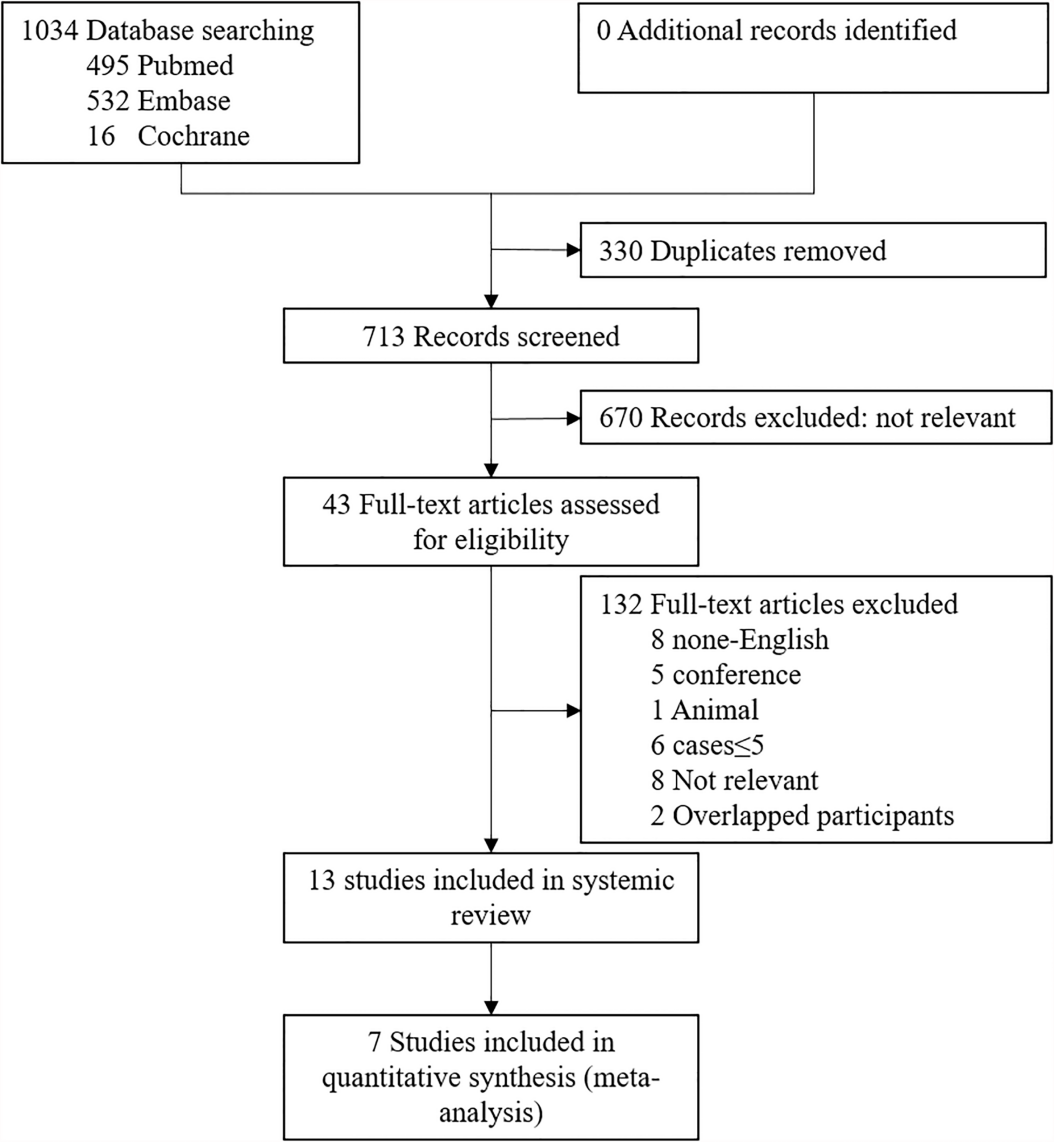
Flow Diagram of the Literature Search.

The initial literature search strategy yielded a total of 1043 studies. After comprehensive screening of abstracts, only 43 articles were included in the full-text review. Of these papers, we excluded 8 non-English articles, 5 conference abstracts, 1 animal study, 6 case studies ≤ 5, 8 irrelevant articles, and 2 studies with overlapping participants. The remaining 13 papers including 302 patients with EP and 270 patients with CP met the eligibility criteria for qualitative synthesis (9, 15–26). Then, 7 studies providing a control group for comparison were included in the final quantitative analysis of outcomes (9, 21–26). A flow diagram of the identification and selection of eligible studies is shown in **Figure 1**.

### Characteristics of Included Studies

In total, 6 of 13 included studies were single-arm (15–20) while others provided a control group for comparison (9, 21–26). Four studies (22–24, 26) adopted prospective design whereas others were retrospective. The earliest included study was published in 2000 and the latest was published in 2020. The included studies were all performed in China and Korea. The latest study involved robotic-assisted parotid surgery (15), while the others were endoscopic assisted surgery. Among the 13 studies, no patients were converted to open surgery and only two patients were found to have tumor recurrence during the follow-up period. The first study used a modified “Blair” incision (20), the other used a transoral approach (22), and the rest used preauricular, retroauricular, hairline, or submandibular incisions. The region of most studies was limited to the superficial lobe of the parotid gland. However, 3 studies (9, 15, 21) were involved in deep lobe lesions while the other 2 studies were concentrated on accessory parotid (16, 22). Characteristics of included studies are shown in **Table 1**. Intraoperative and postoperative parameters of included studies are shown in **Table 2**.

**Table 1:** Characteristics of the included studies.

Study	Year	Country	Study Type	No. (Male/Female)		Age, mean (SD), y		Tumor size, mean (SD), cm		Surgical region		Approach	Follow-up, median, months	
				EP	CP	EP	CP	EP	CP	EP	CP	EP	EP	CP
Park et al.	2020	Korea	R	25/28^a^	NA	39	NA	NR	NA	SL, DL	NA	RAHI	NR	NA
Zhang et al.	2015	China	R	5/8	NA	14.2 (6.3)	NA	2.0×2.6	NA	AP	NA	PA	3-14	NA
Woo et al.	2015	Korea	R	5/13	NA	27.3 (6.6)	NA	2.1×1.8	NA	SL	NA	HI	16	NA
Huang et al	2009	China	R	13/5	NA	17-62	NA	2.5 (0.4)	NA	SL	NA	RA	26-42	NA
Chen et al.	2007	China	R	12/2	NA	41.8	NA	3.9×2.4×1.7^b^	NA	NR	NA	RA	26^c^	NA
Lin et al.	2000	China	R	12/4	NA	40-75	NA	NR	NA	NR	NA	Modified ‘Blair’	NR	NA
Li et al.	2019	China	R	8/7	39/18	53.0 (17.1)	52.5 (15.4)	2.3	3.0	SL, DL	SL, DL	RA	33	33
Kim et al.	2019	Korea	P	11/9	12/10	34.30 (8.61)	36.81 (8.77)	2.80 (0.89)	2.59 (0.81)	AP	AP	Transoral	13.4 (1.27)	14.45 (1.76)
Gao et al.	2019	China	R	17/20	55/32	47.00 (16.97)	51.37 (15.27)	2.35 (0.76)	2.38 (1.11)	SL, DL	SL, DL	RAHI, RM, THI	12	14
Fan rt al.	2017	China	P	14/7	15/10	38.7^b^	43.3	2.7 (1.6)	2.8 (1.9)	SL	SL	RA	25	27
Yan et al.	2015	China	P	9/20	10/19	45.1 (16.4)	45.7 (15.8)	2.8 (1.8)	2.9 (1.0)	SL	SL	RA	3-72	3-72
Chen et al.	2014	China	R	15/15	17/13	48 (11)	47 (12)	2.4 (0.5)	2.5 (0.4)	SL	SL	RA	9-36	9-36
Huang et al	2009	China	P	13/5	14/6	44.22 (16.18)	45.50 (14.17)	2.36 (0.53)	2.43 (0.47)	SL	SL	RA+RM	30	32

P, prospective; R, retrospective, EP, endoscopic-assisted parotidectomy; CP, conventional parotidectomy; SL, superficial lobe; DL, deep lobe; AP, accessory parotid; PA, preauricular; RA, retroauricular; HI, hairline; RAHI, retroauricular and hairline; THI, temporal hairline; RM, retromandibular; NR, not reported; NA, not available.

a Robotic group. One patient underwent parotidectomy and thyroid lobectomy.

b Median.

c Mean.

**Table 2:** Summary of Intraoperative and postoperative parameters.

Study	Year	Operative time mean (SD), min		Incision length mean (SD), cm		Intraoperative bleeding mean (SD), ml		Satisfaction mean (SD)		Complications, No.				Drainage volume mean (SD), ml		Length of stay mean (SD), days		Recurrence No.	
										FN palsy		others							
		EP	CP	EP	CP	EP	CP	EP	CP	EP	CP	EP	CP	EP	CP	EP	CP	EP	CP
Park et al.^a^	2020	272	NA	NR	NA	24	NA	1.1	NA	3	NA	0	NA	152	NA	6.3	NA	NR	NA
Zhang et al.	2015	54	NA	2	NA	4-15	NA	satisfied	NA	0	NA	0	NA	NR	NA	NR	NA	0	NA
Woo et al.	2015	82.5	NA	5.5	NA	minimal	NA	9.77	NA	1	NA	0	NA	NR	NA	NR	NA	0	NA
Huang et al	2009	98.7	NA	3.3	NA	14.7	NA	satisfied	NA	2	NA	0	NA	NR	NA	NR	NA	0	NA
Chen et al.	2007	114	NA	3.1	NA	minimal	NA	satisfied	NA	2	NA	0	NA	NR	NA	NR	NA	1	NA
Lin et al.	2000	NR	NA	6.9	NA	NR	NA	satisfied	NA	0	NA	0	NA	NR	NA	NR	NA	NR	NA
Li et al.^b^	2019	98	115	NR	NR	30	50	0	3	3	8	1	22	35	59	5	6	0	0
Kim et al.	2019	47.5 (9.93)	82.72 (15.86)	3.55 (0.99)	6.40 (1.18)	minimal	minimal	9.66 (0.47)	6.72 (1.80)	1	3	0	6	NR	NR	NR	NR	0	0
Gao et al.	2019	97.84 (23.7)	120.34 (80.95)	4.66 (0.78)	12.98 (1.28)	26.76 (12.2)	65.29 (141.42)	satisfied	NR	8	18	39^c^	49^c^	NR	NR	9.12 (1.12)	11.33 (3.94)	0	1
Fan rt al.	2017	83.1 (21.3)	79.4 (17.5)	3.6 (0.5)	9.1 (1.9)	23.6 (8.9)	90.7 (34.4)	9.1 (1.4)	6.3 (2.6)	2	6	4	10	30.8 (8.7)	54.9 (12.7)	NR	NR	0	0
Yan et al.	2015	141.7 (51.2)	138.1 (34.2)	4.3 (0.5)	9.3 (1.2)	26.6 (10.4)	108.6 (40.2)	8.9 (0.7)	6.7 (1.8)	2	8	2	9	NR	NR	NR	NR	0	0
Chen et al.	2014	74.8 (15.7)	103.2 (10.3)	4.8 (0.4)	12.2 (1.4)	12.7 (3.9)	31.0 (8.9)	8.6 (1.2)	5.4 (1.3)	1	0	0	0	NR	NR	NR	NR	0	0
Huang et al	2009	108.61 (11.86)	105.25 (10.70)	NR	NR	13.89 (3.23)	30.25 (7.86)	satisfied	NR	1	2	1	1	NR	NR	NR	NR	0	0

EP, endoscopic-assisted parotidectomy; CP, conventional parotidectomy; NR, not reported; NA, not available; FN, facial nerve.

a Exclude data of one patient with thyroidectomy.

b Continuous variables were expressed in median.

c Original data were not considered accurate.

### Quality Assessment

Cochrane tool scores for 3 RCTs are shown in **eTable 1** and the MINORS scores are summarized in **eTable 2**. For both researchers and patients in all included studies, the treatment methods were known. Due to insufficient follow-up time to monitor recurrence, all studies did not receive a high score in follow-up items.

### Primary Outcomes

**Figure 2:**
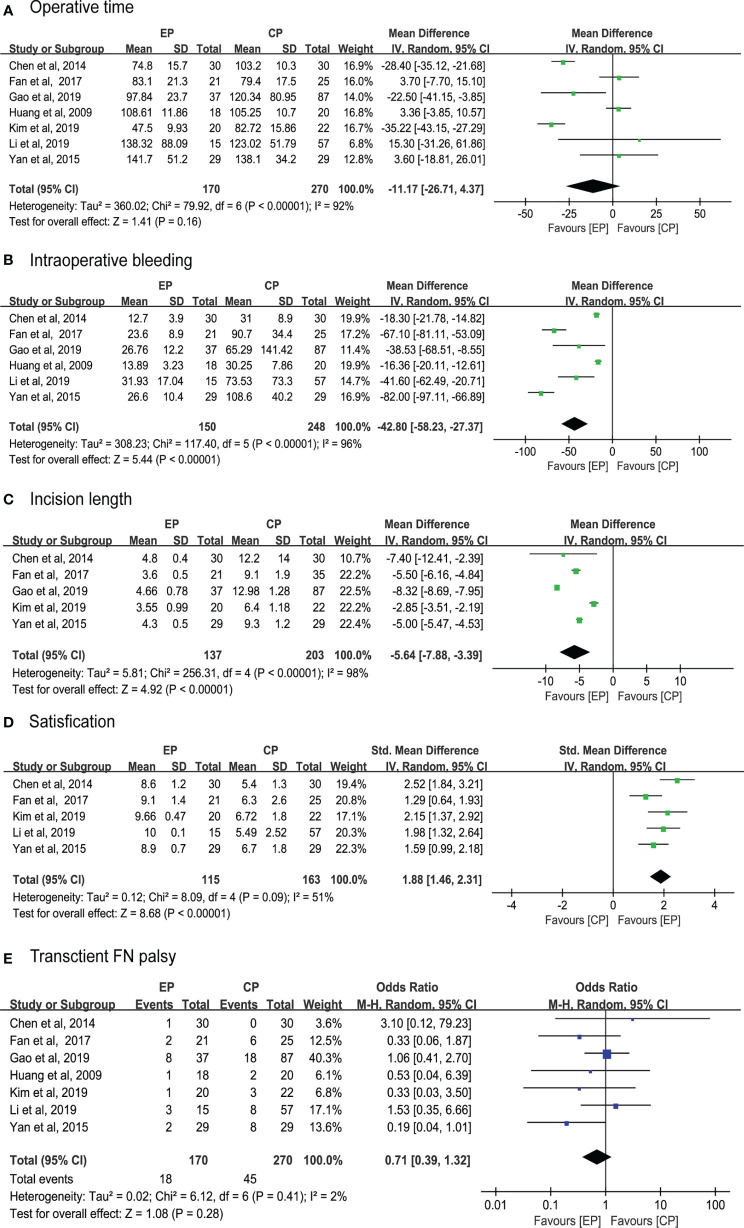
Primary outcome.

A total of 12 studies reported on the operative time of which 7 compared with CP (9, 21–26). Pooled data analysis revealed that operative time was insignificantly different for the EP group compared with the CP group (WMD, 95% CI, -11.17; -26.71 to 4.34; p = 0.16) with high heterogeneity (I^2^ = 92%) (**Figure 2A**).

The intraoperative bleeding volume was reported in 12 studies, of which 7 compared with CP (9, 21–26). However, the authors of three studies provided no numerical data. The results of pooled data analysis were significant for the EP group compared with the CP group (WMD, 95% CI, -42.80; - 58.23 to -27.37; p < 0.01) with high heterogeneity (I^2^ = 96%) (**Figure 2B**).

Incision length was observed in 10 studies in which 5 compared with CP (9, 22–25). Pooled results showed that the incision length was shorter in the EP group (WMD, 95% CI, -5.64; -7.88 to -3.39; p < 0.01) with high heterogeneity (I^2^ = 98%) (**Figure 2C**).

The satisfaction was reported in all included studies, but 6 of which just presented with satisfaction. Compared with the CP group (21–25), pooled data of VAS score was significantly higher in the EP group (SMD, 95% CI, 1.88; 1.46 to 2.31; p < 0.01) with moderate heterogeneity (I^2^ = 51%) (**Figure 2D**).

Facial palsy, as the main complication, was reported in all included studies with a total of 26 cases in the EP group and 45 in the CP group. However, all of those cases were transient and recovered in the follow-up period. Pooled data analysis was insignificantly different for the two method (OR, 95% CI,0.71; 0.39 to 1.32; p = 0.28) with low heterogeneity (I^2^ = 2%) (**Figure 2E**).

### Secondary Outcomes

**Figure 3:**
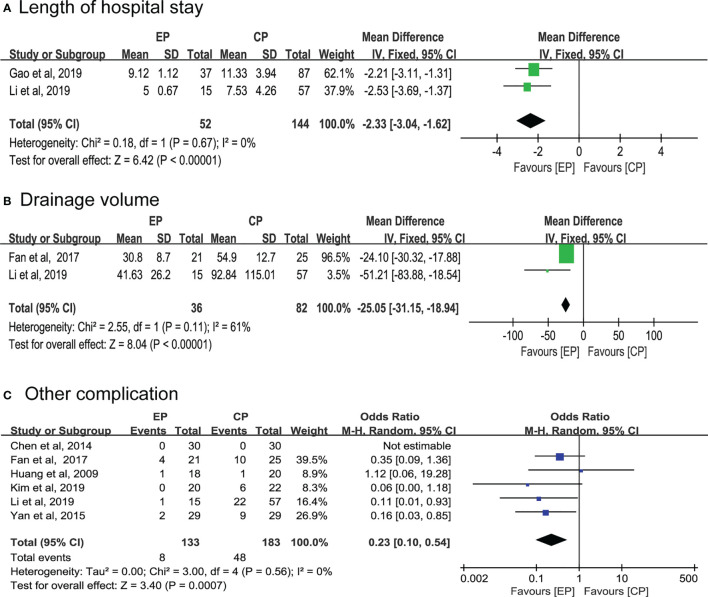
Secondary outcome.

Two articles respectively compared the length of hospital stay and drainage volume between the EP and CP groups (9, 21, 23). Notably, pooled data of both results supported endoscopic group (WMD, 95% CI, -2.33; -3.04 to -1.62; p < 0.01; 95% CI, -25.05; -31.15 to -18.94; p < 0.01; respectively) (**Figures 3A, B**). Additionally, the result indicates that other complications were fewer in the EP group than in the CP group (OR, 95% CI,0.23; 0.10 to 0.54; p < 0.01) (**Figure 3C**).

### Sensitivity Analysis

Considering the difference of surgical approach and the potential error of data conversion, we performed a sensitivity analysis on the primary outcome by removing Kim et al. (22) and Li et al. (21) studies. Our results revealed that the influence of these two studies set on the pooled results was insignificant.

## Discussions

Traditional parotidectomy using Blair incision or its improved incision has been demonstrated to expose all parotid tissues well, but it leaves a 10 cm long incision on the cheek, severely affecting the postoperative aesthetics, especially in patients with scar constitution (27). Recently, endoscopic assisted management has shown good prospects in a variety of head and neck surgeries, such as thyroid surgery, parapharyngeal space surgery, and selective neck dissection, among others. Similarly, the benefits of minimally invasive and magnifying endoscopy are also suitable for parotid surgery in theory. We therefore conducted this review and meta-analysis to systematically introduce the application of endoscopy in parotid gland surgery, as well as evaluate the advantages and disadvantages of this technique compared with traditional parotidectomy.

Our data indicate that the operative time was insignificant in the EP group than in the CP group with high heterogeneity. We observed in different study groups, the operation time of CP was comparable. Compared with the EP group, we noted that the master of CP was high, but the mastery of EP was different. The operation time of EP is expected to be shortened in the future, particularly with the increase in proficiency.

Traditional parotidectomy is usually performed using a Y-shaped or S-shaped incision, with an incision length close to 10 cm. Although new incision designs have been proposed, such as V-shaped periauricular incision, the incision length is often insignificantly shortened and cannot be covered well (4). One approach to overcome this problem is to apply the advantages of the endoscope to shorten the incision length as much as possible. The second is to use the natural masking effect of mastoid hair behind the parotid gland to place the incision in the hairline. Herein, we found the incision length of the EP group was less than 5 cm, while that of the CP group was more than 5 cm. These findings imply that the incision length of the EP group was significantly better than that of the CP group. We also uncovered that although different research groups used different incisions, including preauricular, postauricular, hairline, and oral mucosa, they all achieved good cosmetic effects based on successful completion of the operation and had higher cosmetic satisfaction compared with the CP group. Presently, there is a paucity of published literature that has examined how non-endoscope-assisted parotidectomy is performed through the facelift approach. However, this operation requires more traction and skin flap separation than endoscopically assisted parotidectomy (28).

Facial nerve injury is the most significant complication of parotidectomy. Large institutional series report transient facial nerve dysfunction occurring in up to 65% of parotidectomy patients and permanent facial nerve weakness in approximately 5% of cases (29, 30). In this review, six of the included studies (15, 17, 19, 21–23) used intraoperative nerve detectors, and the final pooled results suggest that there is no significant difference in the incidence of postoperative nerve palsy between EP and CP groups. We strongly believe that although it is difficult to fully expose all surgical fields at one time under endoscopy, with the improvement of endoscopic visualization technology, skilled surgeons can easily expose nerves to show their location and accurately determine their course. Additionally, the nerve detector can be applied for intraoperative nerve protection. Current studies have confirmed that in primary cases of parotidectomy, intraoperative facial nerve monitor decreases the risk of immediate postoperative facial nerve weakness (31). However, in other complications, except facial paralysis, the EP group was significantly better than the control group. Furthermore, the number of postoperative numbness in the EP group was lower than that of the CP group due to small incisions.

In addition to the advantages of EP discussed above, another point that cannot be ignored is the surgical indications. The classic Y-shaped or S-shaped incision can fully expose the parotid gland, which is suitable for the treatment of any parotid gland lesions, including benign and malignant lesions. To achieve the postoperative aesthetic effect, EP has obvious limitations on the surgical indications, mainly including the following points: (1) the size of lesions depends on the length of the incision design; (2) the lesions are limited to the superficial lobe of the parotid gland; (3) benign tumors; and (4) no history of radiotherapy and surgery. Radiotherapy or surgical history can make local tissue adhesion tight, even damage the original location mark, which will significantly increase the difficulty of surgery. Recent studies have shown that malignant tumors of the parotid gland and deep lobe lesions of the parotid gland can significantly increase the difficulty of the operation and the incidence of postoperative facial weakness (32). Therefore, in this study, most researchers excluded such patients before surgery. Li et al. (21) enrolled patients with low-grade T1 and T2 tumors without lymph node metastasis, and endoscopic assisted total parotidectomy was completed with the help of a nerve monitor. However, as the researchers noted, the surgeon must be skilled in the use of a nasal endoscope as well as enriched experience of parotid surgery. Robot-assisted neck lymph node dissection has been reported in many studies (33–35). Park et al. successfully completed parotid gland operation and neck lymph node dissection using robot-assisted technique through the posterior hairline incision, which greatly expanded the indications of parotid surgery and achieved good cosmetic results (15). Nevertheless, limited by the hardware conditions, the popularization of this technology still needs a long time.

### Limitations

There still exist some limitations in this review. First, all included studies were conducted in China and Korea, potentially limiting the generalizability of our findings. Second, some between-study heterogeneity was checked in some comparisons, possibly due to differences in surgical approaches, inaccuracy of data conversion, and surgeon experience. Third, most of the studies included were nonrandomized trials, three studies with randomized design could not be completely blinded due to the nature of the surgery, which might over- or underestimate the measured effect. Fourth, the length of follow-up of the included studies is insufficient, which raises the question of undocumented recurrence.

## Conclusion

Taking into account the above shortcomings and the small sample size, we suggest that the current evidence does not adequately support EP is equally safe and effective as CP. In certain selected cases, endoscopic technology has its unique advantages. For patients with strong cosmetic needs, endoscopic or robotic techniques may be an alternative through adequate preoperative evaluations.

## Data Availability Statement

The original contributions presented in the study are included in the article/**Supplementary Material**. Further inquiries can be directed to the corresponding author.

## Author Contributions

Concept and design: All authors. Acquisition, analysis, or interpretation of data: SC, MZ, and YL. Drafting of the manuscript: SC and MZ. Critical revision of the manuscript for important intellectual content: All authors. Statistical analysis: SC and MZ. Administrative, technical, or material support: All authors. Supervision: JQ and YL. All authors contributed to the article and approved the submitted version.

## Conflict of Interest

The authors declare that the research was conducted in the absence of any commercial or financial relationships that could be construed as a potential conflict of interest.

## Publisher’s Note

All claims expressed in this article are solely those of the authors and do not necessarily represent those of their affiliated organizations, or those of the publisher, the editors and the reviewers. Any product that may be evaluated in this article, or claim that may be made by its manufacturer, is not guaranteed or endorsed by the publisher.
